# Liver Cirrhosis Patients Who Had Normal Liver Function Before Liver Cirrhosis Development Have the Altered Metabolic Profiles Before the Disease Occurrence Compared to Healthy Controls

**DOI:** 10.3389/fphys.2019.01421

**Published:** 2019-11-19

**Authors:** Hye Jin Yoo, Keum Ji Jung, Minkyung Kim, Minjoo Kim, Minsik Kang, Sun Ha Jee, Yoonjeong Choi, Jong Ho Lee

**Affiliations:** ^1^National Leading Research Laboratory of Clinical Nutrigenetics/Nutrigenomics, Department of Food and Nutrition, College of Human Ecology, Yonsei University, Seoul, South Korea; ^2^Research Center for Silver Science, Institute of Symbiotic Life-TECH, Yonsei University, Seoul, South Korea; ^3^Institute for Health Promotion, Graduate School of Public Health, Yonsei University, Seoul, South Korea

**Keywords:** liver cirrhosis, metabolic dysregulation, early biomarkers, Korean cohort, prospective study

## Abstract

Liver cirrhosis (LC) is the final usual outcome of liver damage induced by various chronic liver diseases. Because of asymptomatic nature of LC, it is usually diagnosed at late and advanced stages, and patients are easy to miss the best timing for treatment. Thus, the early detection of LC is needed. In the prospective Korean Cancer Prevention Study-II (K-II), we aimed to identify valuable biomarkers for LC using metabolomics to distinguish subjects with incident LC (LC group) from subjects free from LC (control group) during a mean 7-year follow-up period. Metabolic alterations were investigated using baseline serum specimens acquired from 94 subjects with incident LC and 180 age- and sex-matched LC-free subjects via ultra-performance liquid chromatography (UPLC)-linear-trap quardrupole (LTQ)-Orbitrap mass spectrometry (MS). As a result of the metabolic analysis, 46 metabolites were identified. Among them, 11 and 18 metabolite level showed a significant increase and decrease, respectively, in the LC group compared to the control group. Nine metabolic pathways, including glyoxylate and dicarboxylate metabolism, amino acid metabolism, fatty acid metabolism, linoleic acid metabolism, α-linolenic acid metabolism, and arachidonic acid metabolism, were significantly different between the two groups. Logistic regression demonstrated that the LC emergence was independently affected by serum levels of myristic acid, palmitic acid, linoleic acid, eicosapentaenoic acid (EPA), lysophosphatidic acid (LPA) (18:1), glycolic acid, lysophosphatidylcholine (lysoPC) (22:6), and succinylacetone (*R*^2^ = 0.837, *P* < 0.001). This prospective study revealed that dysregulation of various metabolism had the clinical relevance on the LC development. Moreover, myristic acid, palmitic acid, linoleic acid, EPA, LPA (18:1), glycolic acid, lysoPC (22:6), and succinylacetone were emerged as independent variables influencing the incidence of LC. The results support that the early biomarkers found in this study may useful for predicting and remedying the risk of LC.

## Introduction

Various chronic liver diseases induce liver damage, finally leading to liver cirrhosis (LC) as the final common pathological outcome (Zhou et al., 2014). The cause of LC includes alcoholism, chronic hepatitis B (Asia-Pacific region) or C (western countries) virus infection, and non-alcoholic fatty liver disease (Liaw et al., 2008; Innes et al., 2013). Over early cirrhosis, the liver can compensate the changes leaded by bridging fibrosis, and most patients do not have any special symptoms unless entering the decompensated cirrhosis stage (Qi et al., 2012). Decompensated cirrhosis, also called end-stage liver disease, is a general factor of global mortality; liver disease is the 7th and 12th cause of death in Europe and United States, respectively (Peng et al., 2019). In Korean population (aged 20–65), liver disease is the 8th leading cause of overall death in 2017 (Korean Statistical Information Service [KOSIS], 2018). Because of asymptomatic nature of LC, LC is usually diagnosed at late and advanced stages and patients often miss the best opportunity for therapy. Therefore, for betterment of the diagnosis and prognosis, identifying additional and reliable markers possibly used for early and accurate detection of LC is necessary.

Metabolomics scientifically provides new aspects of disease biomarker by analyzing biological samples and by offering all detectable metabolites (metabolic profiling) (Jasbi et al., 2019). Indeed, for observing metabolic alterations caused by various disease, mass spectrometry-based metabolic profiling is a good analytical platform (Jasbi et al., 2019); and this technology is widely used to discover new biomarkers (Chen et al., 2013; Lu et al., 2015). Researchers have attempted to investigate metabolic profiling of LC, but the results regarding pre-diagnostic biomarkers of LC are contradictory. Furthermore, few studies have focused on prospective settings. Therefore, in the prospective Korean Cancer Prevention Study-II (KCPS-II), which enrolled a cancer-free cohort at baseline, we applied metabolomics to distinguish individuals with LC (LC group) from age- and sex-matched controls who were free of LC (healthy controls) over a mean 7-year follow-up period. Our objective was to identify early biomarkers with value in differentiating these two groups using their baseline serum metabolic profiles, as measured using ultra-performance liquid chromatography (UPLC)-linear-trap quadrupole (LTQ)-Orbitrap mass spectrometry (MS).

## Materials and Methods

### Study Population

Study participants were recruited from the KCPS-II Biobank during routine health check-up at 18 health promotion centers in Seoul and Gyeonggi-do, South Korea (from 2004 to 2013). Detailed information of the KCPS-II is described in a previous publication (Jee et al., 2018). Inclusion criterion was normal levels of liver enzymes, alanine aminotransferase (ALT) and aspartate aminotransferase (AST), which reflect normal liver function and hepatocyte damage, respectively; upper limits of normal ALT were 34 and 24 U/L in men and women, respectively, and upper limits of normal AST were 32 and 26 U/L in men and women, respectively. These values were set according to a previous report for Koreans (Sohn et al., 2013). Exclusion criteria were underweight or overweight/obesity [18 kg/m^2^ ≤ body mass index (BMI) < 25 kg/m^2^]; hypertension (systolic/diastolic blood pressure ≤ 140/90 mmHg); diabetes (fasting glucose ≤ 126 mg/dL); alcohol abuse/alcoholic; or any medication.

Among the 156,701 KCPS-II subjects, who were fully given study explanation and provided written consent, 94 individuals developed LC during the 7-year follow-up period (LC group); at baseline, these individuals had had normal liver function and no diagnostic evidences of LC during the health check-up. At an approximately 1:2 ratio, 180 age- and sex-matched healthy subjects (normal liver function at baseline and did not develop LC during the 7-year follow-up period) were included as a control group in the final analysis. The study, which complied with the principle in the Declaration of Helsinki, was reviewed and approved by the Institutional Review Board of Yonsei University.

### Blood Collection and Biochemical and Anthropometric Assessments

For all assessment, we used baseline serum samples to determine any clinical and biochemical differences between the control and LC groups prior to LC occurrence. For acquiring the serum samples, peripheral venous blood was obtained after a fasting period (minimum 12 h) from each study participants and the serum was separated from the specimens. The serum aliquots were then stored at –70°C prior to further examination.

For biochemical assessments, fasting serum glucose, total cholesterol, triglyceride, low-density lipoprotein (LDL)-cholesterol, high-density lipoprotein (HDL)-cholesterol, AST, ALT, and high-sensitivity C-reactive protein (hs-CRP) levels were assessed via automatic analyzers. Each laboratory measurement was carried out according to the internal and external quality control (QC) procedures specified by the Korean Association of Laboratory QC. The agreement for each measurement across respective hospitals was high (correlation coefficients ranging from 0.96 to 0.99) (Jee et al., 2014).

Regarding the anthropometric assessments, height (cm) and body weight (kg) were measured with light clothing. To calculate BMI, body weight (kg) was divided into height in meter squared (m^2^). Waist circumference (cm) was measured midway between the lower rib and the iliac crest. Systolic and diastolic blood pressures (mmHg) were measured after a rest period of at least 15 min. To gather smoking and alcohol consumption histories, each study participants were interviewed with a structured questionnaire.

### Diagnosis of LC

From the National Health Insurance Service (NHIS), health insurance claim information about LC incident were acquired. Among the data obtained from the NHIS, patients who were hospitalized and outpatients more than once were defined as having LC. LC was coded as K74 according to the International Classification of Diseases, Tenth Revision (ICD-10) (World Health Organization [WHO], 2011).

### Non-targeted (Global) Metabolic Profiling Using Serum

#### Sample Preparation and UPLC-LTQ-Orbitrap MS

Detailed description regarding a preparation of serum samples was reported elsewhere (Jee et al., 2016). Briefly, 100-μL aliquots of the baseline serum samples, obtained during the health check-up, were mixed with acetonitrile (800 μL) by vortexing. The mixtures were centrifuged (10,000 rpm, 5 min, 4°C), and then supernatant was dried with N_2_ gas. After drying process, remaining pallet was dissolved in 10% methanol, vortexed, and centrifuged (10,000 rpm, 5 min, 4°C). The supernatant was then transferred to a vial.

Full information of UPLC-LTQ-Orbitrap MS analysis was also described in our previous research as a Supplementary Material (Yoo et al., 2018). In brief, an Acquity UPLC-BEH-C18 column (2.1 × 50 mm, 1.7 μm; Waters, Milford, MA, United States) was equipped in a Thermo UPLC system (Ultimate 3000 BioRS; Dionex-Thermo Fisher Scientific, Bremen, Germany). The prepared samples were extracted and injected (5 μL) into the column, and liquid chromatographic separation was carried out in both electrospray ionization (ESI)-positive and -negative modes. The metabolites, separated by the column under each ESI-positive and -negative mode, were then applied to an LTQ-Orbitrap MS (Thermo Fisher Scientific, Waltham, MA, United States) operated in full scan mode for Fourier transform MS. The spectra were gathered from *m/z* 50 to *m/z* 1,000. For QC, a pooled QC sample was prepared and injected into every 5th sample. The metabolites’ MS/MS spectra were acquired by applying a collision energy ramping from 20 to 55%.

#### Data Processing and Putative Identification of Metabolites

A SIEVE 2.2 (Thermo Fisher Scientific, Waltham, MA, United States) software was used to process relevant spectral data. Analysis parameters were set as following: retention time width 2.5 min, *m/z* width 5 ppm and *m/z* range 50–1,000. Metabolites were putatively identified by searching based on the databases: ChemSpider^1^, Human Metabolome^2^, KEGG^3^, Lipid MAPS^4^, and MassBank^5^. Selected metabolites were confirmed according to the retention times and mass spectra of standard samples.

### Statistical Analysis

As a statistic analysis tool, a SPSS version 23.0 (IBM/SPSS, Chicago, IL, United States) was utilized. To compare nominal and continuous variables between the two groups, a chi-squared test and an independent *t*-test were conducted. To determine independent effects of each variable on the LC incident, a logistic regression analysis was performed. A two-tailed *P*-values less than 0.05 (*P* < 0.05) was thought to have statistical significance. To avoid errors of multiple comparison with regard to metabolites, *P*-values were adjusted by false discovery rate (FDR); FDR-corrected *q*-values were calculated by a R package “fdrtool” and *q*-values less than 0.05 (*q* < 0.05) were regarded as having a statistical significance.

For a multivariate analysis, a SIMCA-P+ 14.0 (Umetrics Inc., Umeå, Sweden) was used to export spectrometric data. Pareto scaling was used to all data prior to an in-depth analysis. To analyze our models, as a supervised classification tool, orthogonal projection to latent structures-discriminant analysis (OPLS-DA) was used. The validity of the models was evaluated using *Q^2^Y* and *R^2^Y* parameters. A metabolic pathway analysis was conducted by MetaboAnalyst 3.0^6^.

## Results

### Baseline Clinical Characteristics

During the 7 years (mean follow-up period), 94 subjects emerged LC among the 156,701 KCPS-II subjects. The 94 subjects with incident LC were used as the LC group and 180 age- and sex-matched healthy subjects (remained free from LC after the follow-up) were set as the control group. After adjusting for confounding factors (age, sex, BMI, and smoking and drinking status), weight, waist circumference, systolic/diastolic blood pressure, glucose, triglyceride, total cholesterol, HDL-cholesterol, LDL-cholesterol, and hs-CRP did not show any statistical significance between the two groups (Table 1). However, the LC group showed slightly but significantly higher levels of AST (*P* = 0.018), ALT (*P* = 0.030), and γ-glutamyltransferase (GGT) (*P* = 0.022) than the control group. Moreover, the LC group showed a trend toward a decrease in leukocyte number (white blood cell; *P* = 0.057). Additionally, hepatitis B virus surface antigen (HBsAg) and hepatitis C virus antibody (HCV Ab) as one of the causes of LC were assessed at baseline and shown in the Supplementary Table S1.

**TABLE 1:** Clinical characteristics according to onset of liver cirrhosis (LC) in the subjects with normal liver function.

	**Control group**	**LC group**	***P*^*a*^**	***P*^*b*^**
	**(*n* = 180)**	**(*n* = 94)**		
Age (years)	45.2 ± 0.70	43.8 ± 0.99	0.270	–
Male/female *n*, (%)	110 (61.1)/69 (38.3)	57 (60.6)/37 (39.4)	0.763	–
Current smoker *n*, (%)	40 (24.1)	23 (24.5)	0.904	–
Alcohol drinker *n*, (%)	139 (82.7)	67 (71.3)	0.030	–
BMI (kg/m^2^)	23.2 ± 0.20	22.0 ± 0.18	<0.001	–
Weight (kg)	64.6 ± 0.77	60.7 ± 0.86	0.001	0.177
Waist circumference (cm)	79.7 ± 0.67	78.7 ± 0.51	0.007	0.673
Systolic blood pressure (mmHg)	116.2 ± 1.17	113.9 ± 1.08	0.143	0.537
Diastolic blood pressure (mmHg)	72.9 ± 0.78	70.7 ± 0.87	0.073	0.894
Glucose (mg/dL)^∮^	91.7 ± 1.48	87.5 ± 1.15	0.060	0.375
Triglyceride (mg/dL)^∮^	128.8 ± 7.33	110.4 ± 7.26	0.184	0.764
Total cholesterol (mg/dL)^∮^	186.0 ± 2.25	188.6 ± 3.04	0.456	0.260
HDL-cholesterol (mg/dL)^∮^	52.3 ± 0.75	55.4 ± 1.30	0.039	0.241
LDL-cholesterol (mg/dL)^∮^	111.4 ± 2.08	113.8 ± 2.72	0.398	0.205
AST (IU/L)^∮^	19.5 ± 0.27	20.6 ± 0.41	0.022	0.018
ALT (IU/L)^∮^	17.9 ± 0.45	19.0 ± 0.64	0.193	0.030
GGT (IU/L)^∮^	25.1 ± 1.14	27.5 ± 1.86	0.280	0.022
White blood cell (×10^3^/UL)^∮^	5.74 ± 0.12	5.35 ± 0.13	0.035	0.057
hs-CRP (mg/dL)*^∮^*	0.12 ± 0.02	0.20 ± 0.05	0.315	0.518

*Mean ± SE. ^∮^tested by logarithmic transformation. *P*^*a*^-values derived from a chi-squared test and an independent *t*-test for nominal variables and continuous variables, respectively. *P*^*b*^-values derived from an ANCOVA to adjust age, sex, BMI, and smoking and drinking status.*

### Metabolic Profiling in the Serum Samples Using UPLC-LTQ-Orbitrap MS

#### Non-targeted (Global) Metabolic Pattern Analysis

**FIGURE 1:**
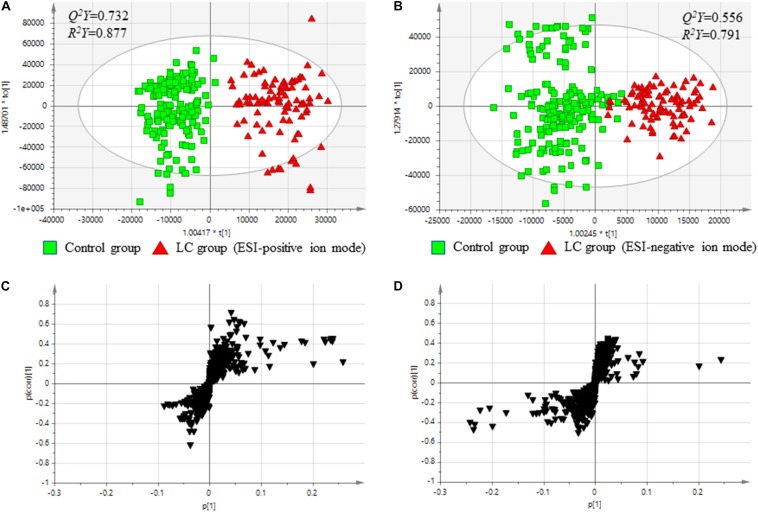
Comparison between serum metabolites in the control and Cirrhosis groups. **(A)** Score plots from OPLS-DA models classifying two groups in positive mode. **(B)** Score plots from OPLS-DA models classifying two groups in negative mode. **(C)** *S*-plots for covariance [p] and reliability correlation [p(corr)] from OPLS-DA models in positive mode. **(D)** *S*-plots for covariance [*p*(1)] and reliability correlation [*p(corr)*(1)] from OPLS-DA models in negative mode. **(A,B)** Comparison between the baseline levels of the control group (free from cirrhosis at baseline and follow-up; *n* = 180) and the baseline levels of the cirrhosis group (free from cirrhosis at baseline and incident cirrhosis at follow-up; *n* = 94).

Using an OPLS-DA score plots, both ESI-positive and -negative ion modes chromatography data on serum metabolites were analyzed. The quality of OPLS-DA was assessed by the values of *Q^2^Y* and *R^2^Y* to evaluate the predictive ability of each model and to confirm that the models were not over-fitted, respectively. Figures 1A,B showed the results of comparisons of baseline metabolite levels between the control and LC groups in ESI-positive and -negative modes, respectively. The OPLS-DA models displayed acceptable predictive ability and well fitted in ESI-positive ion mode (Figure 1A; *Q^2^Y* = 0.732, *R^2^Y* = 0.877) and ESI-negative ion mode (Figure 1B; *Q^2^Y* = 0.556, *R^2^Y* = 0.791).

The results indicated that the two groups could be discriminated by discrepancies in amount of the metabolites. To confirm potential metabolites providing the differences, S-plots of *p*(1) and *p(corr)*(1) were created for the OPLS-DA models using Pareto scaling in ESI-positive and -negative ion modes (Figures 1C,D). The S-plots showed that metabolites having higher or lower values of *p(corr)* were more related to separate between the control and the LC groups.

#### Baseline Serum Metabolites

Peaks (serum metabolites) that importantly discriminate between the control and LC groups were selected in accordance with the variable importance in the projection (VIP) values (VIP ≥ 1.5). As comparing the metabolites, detected from the baseline serum samples, between the two groups, a total of 156 metabolites (both ESI-positive and -negative ion modes) had VIP values more than 1.5; of these, 46 metabolites were putatively identified based on the metabolites’ database, whereas the remaining were novel (Supplementary Figure S1). Table 2 presents the serum metabolites in the groups. In the LC group, 18 metabolites had significantly lower peak intensities than in the control group; glycolic acid (*q* = 1.112 × 10^–11^), myristic acid (*q* = 0.019), 3-hydroxytetradecanedioic acid (*q* = 0.025), α-linolenic acid (*q* = 1.877 × 10^–5^), linoleic acid (*q* = 3.805 × 10^–5^), oleic acid (*q* = 0.025), 9-HOTE (*q* = 0.001), 13S-hydroxyoctadecadienoic acid (*q* = 3.326 × 10^–5^), 9-cis-retinoic acid (*q* = 7.158 × 10^–6^), eicosapentaenoic acid (*q* = 5.311 × 10^–5^), 5-HEPE (*q* = 3.021 × 10^–6^), 5-HETE (*q* = 4.986 × 10^–7^), leukotriene B_4_ (*q* = 3.412 × 10^–4^), 17-HdoHE (*q* = 4.587 × 10^–7^), dehydroepiandrosterone sulfate (*q* = 9.100 × 10^–4^), LPA (16:0) (*q* = 0.005), LPA (18:1) (*q* = 2.097 × 10^–5^), and PE (P-16:0e) (*q* = 4.878 × 10^–5^). On the other hand, compared to the control group, the LC group showed 11 metabolites having significantly higher peak intensities; succinylacetone (*q* = 3.242 × 10^–10^), phenylalanine (*q* = 0.023), tyrosine (*q* = 0.004), indoleacrylic acid (*q* = 2.931 × 10^–6^), tryptophan (*q* = 5.017 × 10^–6^), palmitic acid (*q* = 0.043), palmitoylcarnitine (*q* = 8.624 × 10^–4^), oleoylcarnitine (*q* = 7.053 × 10^–4^), lysoPC (16:0) (*q* = 0.006), lysoPC (18:2) (*q* = 0.017), and lysoPC (22:6) (*q* = 0.026) (Table 2).

**TABLE 2:** Putative identification of serum metabolites according to onset of liver cirrhosis (LC) in the subjects with normal liver function.

***m/z* [M+H]*^†^* [M−H]*^‡^***	**Molecular formula**	**Putatively identified metabolites**	**VIP**	***t-*test**		**Cohen’s *d***	**Change trend**
						
				***P***	***q***		
75.009	C_2_H_4_O_3_	Glycolic acid*^‡^*	7.885	3.109E-13	1.112E-11	–0.821	↓
132.101	C_6_H_13_NO_2_	L-Leucine*^†^*	3.513	0.876	0.267	0.018	↑
159.065	C_7_H_10_O_4_	Succinylacetone*^†^*	2.072	1.352E-11	3.242E-10	2.097	↑
166.086	C_9_H_11_NO_2_	L-Phenylalanine*^†^*	3.803	0.033	0.023	0.257	↑
182.081	C_9_H_11_NO_3_	L-Tyrosine*^†^*	2.486	0.004	0.004	0.340	↑
188.070	C_11_H_9_NO_2_	Indoleacrylic acid*^†^*	1.861	6.461E-07	2.931E-06	0.601	↑
205.096	C_11_H_12_N_2_O_2_	L-Tryptophan*^†^*	4.394	1.210E-06	5.017E-06	0.615	↑
227.202	C_14_H_28_O_2_	Myristic acid*^‡^*	2.385	0.026	0.019	–0.259	↓
247.153	C_12_H_22_O_5_	3-Hydroxydodecanedioic acid*^†^*	1.961	0.123	0.067	–0.172	↓
253.216	C_16_H_30_O_2_	Palmitoleic acid*^‡^*	2.869	0.096	0.056	–0.202	↓
255.233	C_16_H_32_O_2_	Palmitic acid*^‡^*	9.512	0.070	0.043	0.236	↑
275.185	C_14_H_26_O_5_	3-Hydroxytetradecanedioic acid*^†^*	2.186	0.037	0.025	–0.222	↓
279.231	C_18_H_30_O_2_	α-Linolenic acid*^†^*	3.423	5.897E-06	1.877E-05	–0.493	↓
279.233	C_18_H_32_O_2_	Linoleic acid*^‡^*	8.057	1.383E-05	3.805E-05	–0.511	↓
281.249	C_18_H_34_O_2_	Oleic acid*^‡^*	7.365	0.037	0.025	–0.240	↓
283.264	C_18_H_36_O_2_	Stearic acid*^‡^*	3.644	0.803	0.250	0.030	↑
295.226	C_18_H_30_O_3_	9-HOTE*^†^*	2.010	0.001	0.001	–0.355	↓
295.227	C_18_H_32_O_3_	13S-Hydroxyoctadecadienoic acid*^‡^*	2.400	1.167E-05	3.326E-05	–0.477	↓
301.215	C_20_H_28_O_2_	9-cis-Retinoic acid*^†^*	3.109	1.842E-06	7.158E-06	–0.521	↓
303.231	C_20_H_30_O_2_	Eicosapentaenoic acid*^†^*	3.694	2.079E-05	5.311E-05	–0.466	↓
303.232	C_20_H_32_O_2_	Arachidonic acid*^‡^*	4.486	0.497	0.172	–0.078	↓
305.247	C_20_H_34_O_2_	11,14,17-Eicosatrienoic acid*^‡^*	1.945	0.980	0.290	0.003	↑
317.211	C_20_H_30_O_3_	5-HEPE*^‡^*	1.809	6.711E-07	3.021E-06	–0.542	↓
319.227	C_20_H_32_O_3_	5-HETE*^‡^*	3.039	7.857E-08	4.986E-07	–0.589	↓
327.232	C_22_H_32_O_2_	Docosahexaenoic acid*^‡^*	4.894	0.143	0.075	–0.173	↓
329.248	C_22_H_34_O_2_	Docosapentaenoic acid*^‡^*	1.906	0.186	0.090	–0.158	↓
335.222	C_20_H_32_O_4_	Leukotriene B_4_*^‡^*	1.745	1.943E-04	3.412E-04	–0.404	↓
343.227	C_22_H_32_O_3_	17-HdoHE*^‡^*	3.320	7.074E-08	4.587E-07	–0.594	↓
367.157	C_19_H_28_O_5_S	Dehydroepiandrosterone sulfate*^‡^*	1.847	6.909E-04	9.100E-04	–0.394	↓
400.341	C_23_H_45_NO_4_	L-Palmitoylcarnitine*^†^*	1.618	6.438E-04	8.624E-04	0.518	↑
409.236	C_19_H_39_O_7_P	LPA (16:0)*^‡^*	6.033	0.006	0.005	–0.325	↓
426.356	C_25_H_47_NO_4_	Oleoylcarnitine*^†^*	2.161	4.898E-04	7.053E-04	0.498	↑
433.236	C_21_H_39_O_7_P	LPA (18:2)*^‡^*	2.276	0.997	0.293	−4.360E-4	↓
435.251	C_21_H_41_O_7_P	LPA (18:1)*^‡^*	3.459	6.726E-06	2.097E-05	–0.550	↓
436.283	C_21_H_44_NO_6_P	PE (P-16:0e)*^‡^*	1.814	1.873E-05	4.878E-05	–0.467	↓
468.307	C_22_H_46_NO_7_P	LysoPC (14:0)*^†^*	1.765	0.127	0.069	0.195	↑
480.343	C_24_H_50_NO_6_P	LysoPC (P-16:0)*^†^*	2.471	0.404	0.146	0.116	↑
494.322	C_24_H_48_NO_7_P	LysoPC (16:1)*^†^*	2.040	0.760	0.240	0.037	↑
496.338	C_24_H_50_NO_7_P	LysoPC (16:0)*^†^*	6.143	0.007	0.006	0.351	↑
510.353	C_25_H_52_NO_7_P	LysoPC (17:0)*^†^*	1.781	0.429	0.153	0.106	↑
518.322	C_26_H_48_NO_7_P	LysoPC (18:3)*^†^*	1.620	0.361	0.134	–0.102	↓
520.338	C_26_H_50_NO_7_P	LysoPC (18:2)*^†^*	3.481	0.023	0.017	0.298	↑
522.353	C_26_H_52_NO_7_P	LysoPC (18:1)*^†^*	2.949	0.281	0.116	0.137	↑
524.369	C_26_H_54_NO_7_P	LysoPC (18:0)*^†^*	3.716	0.838	0.259	0.027	↑
544.338	C_28_H_50_NO_7_P	LysoPC (20:4)*^†^*	1.512	0.170	0.085	0.175	↑
568.337	C_30_H_50_NO_7_P	LysoPC (22:6)*^†^*	1.831	0.039	0.026	0.303	↑

**^†^*Metabolites obtained by ESI-positive ion mode (M/Z [M+H]). *^†^*Metabolites obtained by ESI-negative ion mode (M/Z [M-H]). VIP is variable important in the projection. *P*-values derived from an independent *t*-test between groups; control group (*n* = 180) and LC group (*n* = 94). *q*-Value is adjusted *p*-value that controls the false discovery rate (FDR). Cohen’s *d* is an effect size for the comparison between two means; difference between two means divided by a pooled standard deviation; it is defined as “small, *d* = 0.20,” “medium, *d* = 0.50,” and “large, *d* = 0.80,” Change trend is determined by a comparison of the metabolites’ peak intensities in the LC group with those in the control group.*

### Metabolic Pathways Regarding the LC Pathogenesis in Healthy Subjects

**FIGURE 2:**
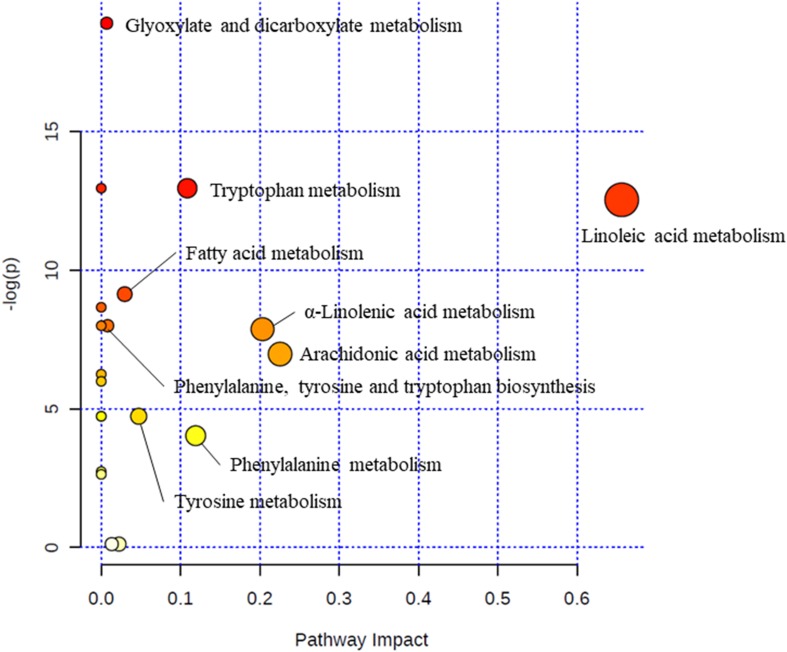
Metabolic pathway analysis. The “metabolome view” presents pathways arranged according to the scores based on enrichment analysis (*y* axis) and topology analysis (*x* axis). The color and size of each circle is based on *P*-values and pathway impact values, respectively.

Via MetaboAnalyst 3.0, a web-based analysis module (see text footnote 6), a metabolic pathway analysis was carried out to verify the proper pathways associated with the selected metabolites (Figure 2). Glyoxylate and dicarboxylate metabolism (glycolic acid; impact = 0.007), tryptophan metabolism (tryptophan; impact = 0.109), linoleic acid metabolism (linoleic acid and 13S-hydroxyoctadecadienoic acid; impact = 0.656), fatty acid metabolism (palmitoylcarnitine and palmitic acid; impact = 0.030), phenylalanine, tyrosine and tryptophan biosynthesis (tryptophan, tyrosine, and phenylalanine; impact = 0.008), α-linolenic acid metabolism (α-linolenic acid; impact = 0.203), arachidonic acid metabolism (5-HETE, leukotriene B4, and arachidonic acid; impact = 0.226), tyrosine metabolism (tyrosine; impact = 0.047), phenylalanine metabolism (tyrosine and phenylalanine; impact = 0.119) were relevant to incidence of LC (Table 3).

**TABLE 3:** Metabolic pathway analysis.

**Metabolic pathways**	**Hits**	***P***	**FDR**	**Impact**
Glyoxylate and dicarboxylate metabolism	Glycolic acid	6.11.E-09	1.22.E-07	0.007
Tryptophan metabolism	L-Tryptophan	2.36.E-06	1.57.E-05	0.109
Linoleic acid metabolism	Linoleic acid	3.58.E-06	1.79.E-05	0.656
	13S-Hydroxyoctadecadienoic acid			
Fatty acid metabolism	L-Palmitoylcarnitine	1.08.E-04	4.31.E-04	0.030
	Palmitic acid			
Phenylalanine, tyrosine and tryptophan biosynthesis	L-Tryptophan	3.37.E-04	8.43.E-04	0.008
	L-Tyrosine			
	L-Phenylalanine			
α-Linolenic acid metabolism	α-Linolenic acid	3.82.E-04	8.49.E-04	0.203
Arachidonic acid metabolism	5-HETE	0.001	0.002	0.226
	Leukotriene B_4_			
	Arachidonic acid			
Tyrosine metabolism	L-Tyrosine	0.009	0.012	0.047
Phenylalanine metabolism	L-Tyrosine	0.018	0.022	0.119
	L-Phenylalanine			

*Hits: the actually matched metabolites uploaded in MetaboAnalyst 3.0. *FDR*: the *P-*values adjusted using false discovery rate (FDR). Impact: the pathway impact value calculated from pathway topology analysis.*

### Logistic Regression Analysis

For evaluating independent influence of following variables on LC incident, logistic regression analysis was performed: AST, ALT, GGT, and 29 metabolites with *q*-values less than 0.05. In all study participants, the incidence of LC was affected by myristic acid, palmitic acid, linoleic acid, eicosapentaenoic acid, LPA (18:1), glycolic acid, lysoPC (22:6), and succinylacetone (*R^2^* = 0.837, *P* < 0.001).

## Discussion

In present-day society, discovering biomarkers of LC is one of the valuable goal to decrease mortality related to liver disease. Although researchers have attempted to find LC-related biomarker, there are still lack of prospective studies to identify early biomarker of LC before its incident. In this study, total 29 metabolites showed the significant differences between the control and LC groups at baseline (before LC incident); 11 and 18 metabolites had significantly increased and decreased peak intensities in the LC group, respectively, compared to the control group. In addition, through the metabolomics technology and metabolic pathway analysis, the present prospective study suggested that deregulation of nine metabolic pathways showed the clinical relevance on LC occurrence in subjects with normal liver function and free from LC at baseline. Thus, these metabolic processes’ alterations are possibly a potential underlying mechanism on LC development. To discuss the metabolites involved in the metabolic alteration regarding incident of LC, we classified them by their type.

### Amino Acids

In the present study, higher levels of phenylalanine, tyrosine, and tryptophan in the LC group in comparison with the control group definitely showed the alteration of amino acid metabolism. These three amino acids are a member of aromatic amino acids (AAAs) and they have been reported to increase in the serum of hepatocellular carcinoma and LC patients (Gao et al., 2009; Fages et al., 2015). While, branched chain amino acids (BCAAs), including leucine, isoleucine, and valine have been informed to decrease in the same condition, thus, an imbalance of the amino acids has been revealed under the hepatocellular carcinoma and LC condition (Gao et al., 2009; Fages et al., 2015). However, the present metabolomics study showed that serum levels of BCAAs were not significantly different between the control and the LC groups. These findings may support the following pathologic progression of LC: initially, BCAAs and AAAs are increased via altered amino acid metabolism; and then, eventual degradation of BCAAs may be occurred by carnitines, which oxidizes BCAAs (Hoppel, 2003).

### Acylcarnitines

Acyl group arising from the metabolism of BCAAs has been suggested to be contributing proportionately to the acyl-carnitine fraction in cirrhosis (Fuller and Hoppel, 1983). During fatty acid oxidation, acylcarnitines are generated as intermediates; and their accumulation is caused by metabolic dysfunctions as a consequence of the insufficient integration between β-oxidation and Krebs cycle (Al-Bakheit et al., 2016). Long-chain acylcarnitines accumulate during certain conditions including hepatic cirrhosis (Fuller and Hoppel, 1983). Additionally, a new pilot study revealed that in patients with hepatocellular carcinoma, serum acylcarnitines had high concentration compared to normal subjects (Yaligar et al., 2016). In this study, even before LC development, the LC group showed higher levels of palmitoylcarnitine and oleoylcarnitine than the control group. Both palmitoylcarnitine and oleoylcarnitine are long-chain acylcarnitines formed from long-chain fatty acid [palmitic acid (C16:0) and oleic acid (C18:1), respectively,] (Kuhajda et al., 1994), therefore, our results are in agree with the studies previously mentioned.

### Saturated Fatty Acids

In this study, in comparison with the control group, the levels of palmitic acid (C16:0) and myristic acid (C14:0) were higher and lower in the LC group, respectively. Palmitic acid (C16:0) is reported to induce lipoapoptosis in hepatocytes via mechanisms, including mitochondrial dysfunction and stress of endoplasmic reticulum (Martínez et al., 2015). Myristic acid (C14:0), which is more rapidly metabolized (both β-oxidation and elongation) in hepatocyte than palmitic acid (C16:0) (Rioux et al., 2000), has been known not to be lipotoxic unlike palmitic acid (C16:0); however, a recent study reported a synergistic effect of myristic acid (C14:0) in palmitic acid (C16:0)-induced lipotoxicity (Martínez et al., 2015). In the present study, lower levels of myristic acid (C14:0) observed in the LC group might be due to its rapid metabolism in hepatocyte; and the rapid metabolism might contribute to accumulation of palmitic acid (C16:0) in the LC group. Furthermore, the logistic regression analysis revealed the independent effect of myristic acid (C14:0) and palmitic acid (C16:0) on LC progression and metabolic pathway analysis showed that palmitic acid (C16:0) involved in fatty acid metabolism which altered in the LC group. These findings, therefore, suggest that early changes in these fatty acids need to be observed to prevent LC development.

### Polyunsaturated Fatty Acids

α-Linolenic acid (C18:3, ω-3 PUFA) is a major precursor of ω-3 PUFA metabolites. In this study, the levels of α-linolenic acid (C18:3) and its downstream ω-3 PUFA metabolites, including eicosapentaenoic acid (C20:5), docosapentaenoic acid (C22:5), and docosahexaenoic acid (C22:6), were lower in the LC group than the control group; however, docosapentaenoic acid (C22:5) and docosahexaenoic acid (C22:6) did not show significant differences. Studies have shown that reduction of circulating levels of ω-3 PUFAs is observed in cirrhotic patients (Watanabe et al., 1999; Ristić-Medić et al., 2013; Enguita et al., 2019). In addition, especially, depletion of docosahexaenoic acid (C22:6) may influence on homeostasis of liver tissue, likely fibrosis progression (Enguita et al., 2019). In the present study, the reason why the LC group had lower levels of ω-3 PUFAs than the control group was hard to know because of limited information. Factors of ω-3 PUFAs deficient could be diverse. First of all, ω-3 PUFAs are essential fatty acids which cannot be synthesized in the body and therefore have to be consumed from diet; thus, a lack of ingestion of ω-3 PUFAs may be a potential reason. Other factors, including malabsorption and changes in microbiota, also can be a reason related to altered circulation levels of ω-3 PUFAs (Enguita et al., 2019). Conclusionally, since the LC group showed a decrease of ω-3 PUFA levels even before LC incident, our results suggest that low levels of ω-3 PUFAs should be carefully observed for preventing future risk of LC development.

In case of ω-6 PUFA, the LC group also showed a significant decrease in linoleic acid (C18:2) compared to the control group. The levels of 13S-hydroxyoctadecadienoic acid, which is a lipoxygenation product derived from linoleic acid (C18:2) (The Human Metabolome Database [HMDB], 2019), was also significantly lower in the LC group. Meanwhile, the levels of arachidonic acid (C20:4), converted from linoleic acid (C18:2), did not show any statistical difference between the groups. There are conflicting reports on the levels of ω-6 PUFAs. A study revealed that arachidonic acid (C20:4) levels showed marked decrease in patients with hepatitis, cirrhosis, and liver cancer compared to healthy controls (Safaei et al., 2016). On the other hand, high ω-6 PUFA to ω-3 PUFA ratio induced by large amount of consumption of ω-6 PUFAs is observed in liver diseases (Wree et al., 2013); and a study demonstrated that high intake of ω-6 PUFAs should be avoid for mitigating progression of liver disease due to their pro-inflammatory characteristics (Ullah et al., 2019). The LC group of this study had low levels of ω-6 PUFAs rather than the control group before LC development; in addition, the inflammatory marker, hs-CRP, was not statistically different between the groups. Thus, inflammation caused by ω-6 PUFAs may not be a principal reason of future LC progression in this study.

### Arachidonic Acid (C20:4) and Eicosapentaenoic Acid (C20:5)-Related Metabolites

5-HETE and leukotriene B_4_ are metabolites associated with arachidonic acid (C20:4) metabolism. Arachidonic acid (C20:4) released by phospholipases from cell membrane is metabolized by lipoxygenases (LOX); especially, 5-LOX generates 5-HETE and leukotriene B_4_ from arachidonic acid (C20:4) (Harizi et al., 2008). These two metabolites induce chemotactic response and chemokinesis of leukocytes and show increased levels in inflammatory lesions for immune function, therefore, 5-HETE and leukotriene B_4_ may play a key role on leukocyte function (Goetzl et al., 1982). In the present study, leukotriene B_4_ and 5-HETE levels were significantly lower in the LC group than the control group and the leukocyte number in the LC group had a trend toward decrease compared to the control group. This result corresponds to a previous research that LC subjects showed significant downregulated levels of eicosanoids including leukotriene in comparison with healthy controls (Fitian et al., 2014). Thus, relatively low leukocyte number, which may be associated with low levels of 5-HETE and leukotriene B_4_, may lead to an improper inflammatory/immune function against future LC progression.

Meanwhile, 5-HEPE is also a metabolite processed by 5-LOX from eicosapentaenoic acid (C20:5) (Onodera et al., 2017). A study demonstrated that eicosapentaenoic acid (C20:5) and 5-HEPE have anti-inflammatory effects on chronic inflammation disease including hepatic steatosis by enhancing anti-inflammatory immune cells, such as T regulatory cells (Onodera et al., 2017). In addition, 5-HEPE lessens inflammatory reaction of macrophage through the JNK pathway and has a protection effect against hepatic steatosis (Wang et al., 2017). Therefore, these metabolites can be considered as an effective factor for preventing early stage of liver disease (before progression of LC). In the present study, both eicosapentaenoic acid (C20:5) and 5-HEPE were significantly lower in the LC group than the control group; moreover, the former was revealed as an independent factor for incidence of LC. Thus, eicosapentaenoic acid (C20:5) and 5-HEPE may become a potent biomarker for prediction LC development.

### LysoPCs

LysoPC is a metabolite released from phospholipids by phospholipase A_2_, an enzyme that involves in hydrolysis the ester bond of *sn*-2 position in phospholipids (Kokotou et al., 2017). This metabolite has been reported to be associated with oxidative stress and inflammation (Zhang et al., 2019), which are related to a progression of liver diseases; oxidative stress has been well known to play important role in liver diseases and chronic inflammation also has been revealed to result in liver fibrosis, cirrhosis, and hepatocellular carcinoma by causing persistent liver injury (Yuan et al., 2019). Indeed, many studies proved that lysoPCs are connected with liver damage. Kakisaka et al. (2012) demonstrated that lysoPCs lead to hepatocyte lipoapoptosis that is germane to liver diseases via various mechanism. Other studies showed that greater concentration of lysoPC in hepatic tissue are observed in non-alcohol steatohepatitis patients compared to healthy controls (Puri et al., 2007; Han et al., 2008). Finally, Yang et al. (2014) showed that lysoPCs were identified as a potential biomarker for early diagnosis of liver fibrosis and cirrhosis. In the current study, the levels of lysoPCs (16:0, 18:2, and 22:6) were detected to be significantly higher in the LC group compared to the control group at baseline. Therefore, our results can support the role of lysoPC as an early biomarker for LC.

From lysoPC species, lysophosphatidic acids (LPAs) are generated by enzymes, autotaxin (ATX) which has lysophospholipase D activity (Kremer et al., 2010). Several studies have been demonstrated that ATX-LPA axis is an important pathological pathway regarding liver diseases (Nakagawa et al., 2011; Erstad et al., 2017; Valdés-Rives and González-Arenas, 2017). In addition, a study showed that elevated serum levels of ATX are positively associated with the severity of LC; and the ATX has a positive correlation with LPA levels in liver cirrhotic patients (Pleli et al., 2014). Unlikely these studies, however, our data showed decreased levels of LPA (16:0 and 18:1) in the LC group. This discordance may come from disease status of individuals. In this study, the time point at which biological samples obtained of the LC group was before LC occurrence, whereas the studies mentioned previously measured the markers under disease existence. Perhaps, the increase of lysoPC levels may precede the LPA elevation in a pathologic progression of LC. Moreover, lysoPC (22:6) and LPA (18:1) were revealed as independent factors for future LC incident via logistic regression analysis. Thus, lysoPC increase and LPA decrease may need to be carefully observed before LC incident.

### Other Metabolites

Glycolic acid and succinylacetone were also revealed as independent factors associated with the incidence of LC in the present study. In the liver, glycolic acid is generated from ethylene glycol by enzymes, alcohol dehydrogenase and aldehyde dehydrogenase; and then is metabolized to oxalic acid by enzymes, glycolate oxidase and glycolate dehydrogenase. Eventually, oxalic acid is excreted in urine via kidney (Corley et al., 2005). Glycolic acid and oxalic acid have been reported to cause metabolic acidosis and acute renal failure (Yamamoto et al., 2011; Tuero et al., 2018). Although our study detected glycolic acid in the LC group, the levels were lower than the control group and their clinical relevance on the liver has not been focused so far. Thus, an attempt to elucidate relationships between glycolic acid and future LC development is needed.

Succinylacetone is a metabolite of tyrosine and has liver toxicity. Fumarylacetoacetate hydrolase (FAH) plays a key role in tyrosine metabolism to produce succinylacetone; and FAH deficiency mainly induced by a heredity problem of FAH encoded gene leads to type 1 tyrosinemia, in which increased levels of succinylacetone are observed along with liver diseases including hepatic failure, LC, and liver cancer (Scott, 2006; Yang et al., 2017). In addition to succinylacetone, levels of tyrosine, methionine, and phenylalanine also have been reported to increase in genetic deficiency of FAH-induced type 1 tyrosinemia (Scorza et al., 2014). Although, in the present study, there were no participants with congenital type 1 tyrosinemia caused by *FAH* gene abnormality, not only succinylacetone but also tyrosine and phenylalanine levels showed significant elevated levels in the LC group compared to the control group. There are limits to know an exact mechanism with our information, however, we suggest a possibility based on our findings: activities of tyrosine metabolism-related enzymes may be altered before LC development.

The present study has limitations. First, even though we obtained the information of LC occurrence from the NHIS record (K74 according to the ICD-10), an exact cause of LC in each subject is still ambiguous; because we did not directly review the patients’ charts and carry out standardized test for LC diagnosis. In addition, depending on which cause or underlying disease leaded to the onset of LC, the metabolic profile at baseline might be possible to vary slightly. Nonetheless, in this study, LC was a final phenotype after all; accordingly, our data can warn about a future risk of LC development. Second, notably, in this study, the exposure assessment was performed at a single time point (baseline; before LC incident) and changes which might be occurred during the follow-up were not measured although the follow-up period was long (7 years); therefore, exact mechanisms on future LC development cannot be fully elucidate with our metabolomics data interpretation. Third, an external validation of our result with an independently different cohort should have performed; however, due to the limitation of available resources (funding, samples, time, etc.), it could not be possible. Instead, we have reviewed carefully our metabolomics data to support their pathological relevance on LC development. Lastly, diurnal variations might exist among the study subjects because sample collection time was varied (a.m. or p.m.). For remedying the shortcomings, further study is needed.

## Conclusion

Despite these limitations, still, many studies can support our results and vice versa. Similar to previous metabolomics studies, the clinical relevance of deregulations of amino acid metabolism, linoleic acid metabolism, fatty acid metabolism, α-linolenic acid metabolism, and arachidonic acid metabolism were observed. In addition, myristic acid, palmitic acid, linoleic acid, eicosapentaenoic acid, LPA (18:1), glycolic acid, lysoPC (22:6), and succinylacetone were revealed as the independent variables related to the future LC development. Metabolic patterns found in this study before LC progression provide meaningful and potential biomarkers for future LC development. The results can provide pathological insight of LC incident and the biomarkers may be useful in early diagnosis of LC.

## Data Availability Statement

The datasets generated for this study are available on request to the corresponding author.

## Ethics Statement

The study participants were fully given study explanation and provided written consent. The Institutional Review Board of Yonsei University reviewed and approved the study, which complied with the principles in the Declaration of Helsinki.

## Author Contributions

HY analyzed and interpreted the data, prepared the manuscript, and contributed to critical revision of the manuscript. KJ acquired the data, provided the blood samples, and prepared the manuscript. MkK, MjK, and MsK analyzed and interpreted the data. SJ provided the blood samples and research funding. YC provided the blood samples. JL interpreted the data, and prepared the manuscript. All authors contributed to the conception and design of the study and minor revision of the manuscript, and have approved the final version of the manuscript for the publication.

## Conflict of Interest

The authors declare that the research was conducted in the absence of any commercial or financial relationships that could be construed as a potential conflict of interest.

## Footnotes

1 http://www.chemspider.com/

2 http://www.hmdb.ca/

3 http://www.genome.jp/kegg/

4 http://www.lipidmaps.org/

5 http://www.massbank.jp/

6 http://metaboanalyst.ca/
