# Supplementary material for: Liver Cirrhosis Patients Who Had Normal Liver Function Before Liver Cirrhosis Development Have the Altered Metabolic Profiles Before the Disease Occurrence Compared to Healthy Controls
Source: Front Physiol. 2019 Nov 19;10:1421. doi: 10.3389/fphys.2019.01421 (PMC6877605; doi:10.3389/fphys.2019.01421)
Supplement: Supplementary file 1 [file Table_1.pdf]

Liver cirrhosis patients who had normal liver function before liver cirrhosis development have the altered metabolic profiles before the disease occurrence compared to healthy controls

Supplementary data

Table S1. Results of hepatitis virus test in the study subjects at baseline

	**Control group (*n*=180)**	**LC group (*n*=94)**	***P***
**HBsAg *n*, (%)**			
Carriers	7 (16.7)	35 (27.2)	<0.001
Non-carriers	158 (72.8)	59 (83.3)	
**HCV Ab *n*, (%)**			
Carriers	- (-)	2 (2.9)	0.040
Non-carriers	146 (68.2)	68 (97.1)	

P-values derived from a chi-squared test. HBsAg: hepatitis B virus surface antigen. HCV Ab: Hepatitis C virus antibody. In the control group, 15 and 34 subjects did not have the information of HBsAg and HCV Ab tests, respectively (missing data). In the LC group, 24 subjects did not have the information of HCV Ab tests (missing data).

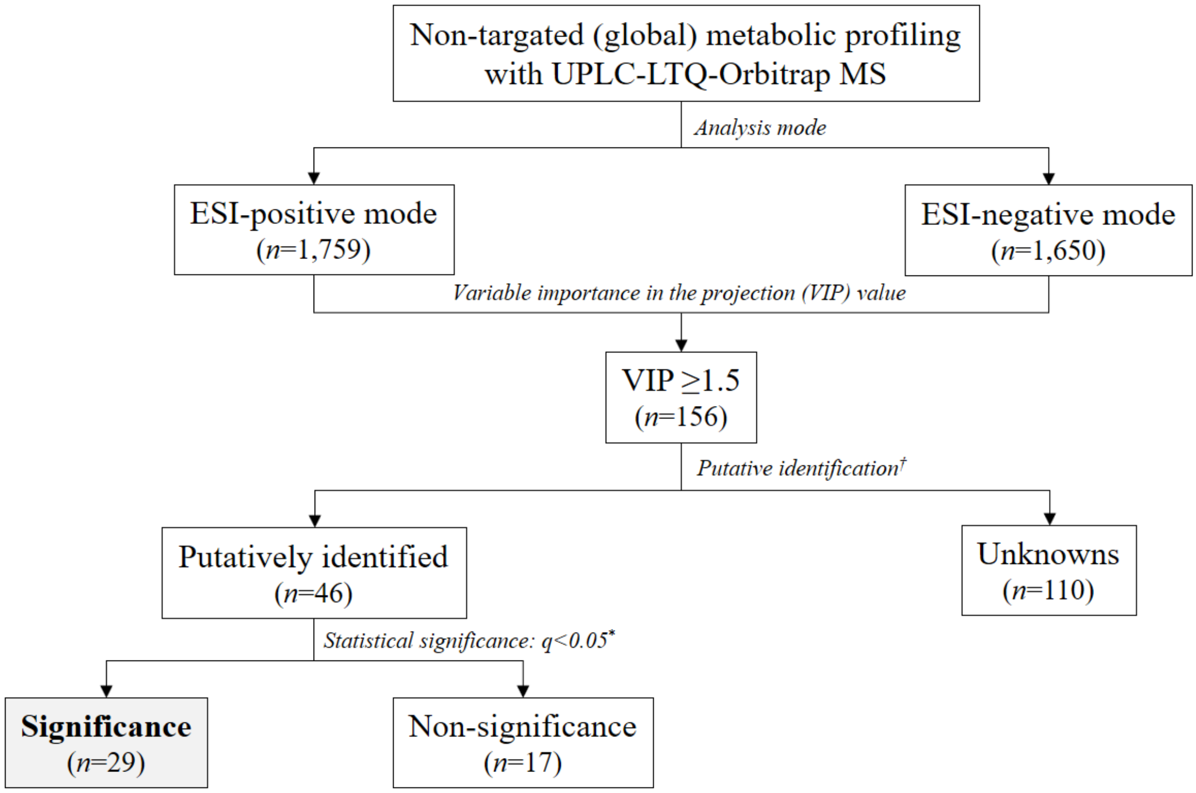

Figure S1. Flow chart on the selection of the significant metabolites

Serum metabolites were analyzed using the non-targeted (global) metabolic profiling (UPLC-LTQ-Orbitrap MS). Metabolites' separation was carried out in both electrospray ionization (ESI)-positive and -negative modes. All detected metabolites were screened out by a VIP value; VIP ≥1.5. †Putative identification: metabolites were putatively identified by searching based on the database (ChemSpider, Human Metabolome, KEGG, Lipid MAPS, and MassBank). *q-Value is an adjusted P-value that controls the false discovery rate (FDR).
